# Gender Minority Stress and Depressive Symptoms in Transitioned Swiss Transpersons

**DOI:** 10.1155/2018/8639263

**Published:** 2018-04-19

**Authors:** Tiziana Jäggi, Lena Jellestad, Salvatore Corbisiero, Dirk J. Schaefer, Josef Jenewein, Andres Schneeberger, Annette Kuhn, David Garcia Nuñez

**Affiliations:** ^1^Department of Psychology, University of Zurich, Binzmühlestrasse 14, 8050 Zurich, Switzerland; ^2^Department of Psychiatry and Psychotherapy, University of Zurich, University Hospital Zurich, Rämistrasse 100, 8091 Zurich, Switzerland; ^3^Division of Clinical Psychology and Psychiatry, University of Basel, Wilhelm Klein-Strasse 27, 4002 Basel, Switzerland; ^4^Department of Plastic, Reconstructive, Aesthetic and Hand Surgery, University of Basel, Basel University Hospital, Spitalstrasse 21, 4031 Basel, Switzerland; ^5^Psychiatric Services Graubünden, Loestrasse 220, 7000 Chur, Switzerland; ^6^Department of Obstetrics and Gynecology, University Hospital Bern, Effingerstrasse 102, 3010 Bern, Switzerland; ^7^Center for Gender Variance, University of Basel, Basel University Hospital, Spitalstrasse 21, 4031 Basel, Switzerland

## Abstract

Compared to the general population, transpersons are exposed to higher levels of discrimination and violence. The stigmatization of transpersons can lead to physical and psychological problems. In particular, transindividuals exhibit a higher prevalence of depression compared to the cispopulation. The gender minority stress model (GMSM) provides a comprehensive theoretical basis to interpret these biopsychosocial interactions. Using the GMSM, this study aimed to identify associations between experience of stigmatization and the mental health of transitioned transpersons using correlational analyses and multiple regression models. In total, 143 transpersons were recruited. Multivariate analyses identified three variables (i.e., unemployment, nonaffirmation of gender identity, and internalized transphobia) to explain variance of depressive symptoms. Furthermore, a mediation of the proximal factors between distal factors and depressive symptoms was found. However, the moderating effect of resilience factors was not demonstrated. The results confirmed the importance of distal and proximal minority stressors for the mental health of transpersons. At the same time, the protective influence of resilience factors seemed to be surprisingly minor. In the treatment of transpersons, practitioners should not only focus on somatic aspects, but also consider the person's previous experiences of stigmatization.

## 1. Introduction

In recent years, there has been an increase in the number of studies reporting a growing transpopulation [1]. For practitioners, the rise of the visibility of transpersons is reflected in a higher demand for medical transition interventions such as gender affirming interventions (GAI) [2]. By reducing the gender dysphoria of transpeople, these medical procedures also contribute secondarily to improving their mental health and quality of life [3]. Even though the risk of developing psychiatric problems decreases after initiation of gender reassignment measures [4], depressive disorders [5] and suicidality [6] are especially discrepantly increased in the transpopulation in comparison to the cispopulation. Other studies have also found higher prevalence of substance use and abuse [7] as well as posttraumatic stress disorder [8].

On a daily basis, transpersons are subjected to stigmatization due to normative gender conceptions in society [9–12]. Experiences of exclusion can take place on structural (e.g., through institutional laws and practices), interpersonal (e.g., through victimization), or personal (e.g., through negative feelings towards one's identity) level [13]. Stigmatization is ubiquitous and can also be found in the healthcare sector. The treatment barriers and the discriminations on the part of the practitioner with which many transpersons are confronted provide examples of structural and interpersonal stigmatization within the various medical care systems [14]. The relationship between stigma, mental health, and the quality of life of transpersons also has clinical implications for those practitioners who care for this group before, during, and especially after medical transition [15]. Thus, there are situations where transpeople do want to undergo a specific GAI (e.g., genital operation and facial feminization), not mainly due to an existing intrinsic gender dysphoria, but primarily to avoid the extrinsic consequences of persistent stigmatization. Without knowledge of these relationships and interactions, there is hardly enough preoperative counselling and education on transpatients, which in turn is likely to affect the degree of satisfaction with the completed surgery.

**Figure 1:**
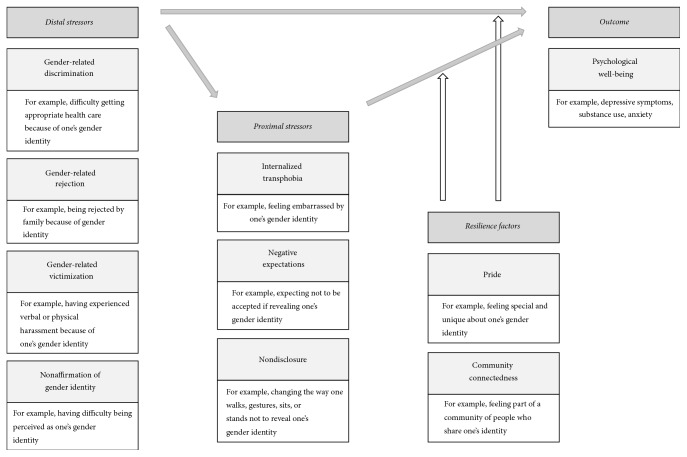
Gender minority stress model as proposed by Testa et al. [17] with distal stressors having a negative effect on psychological well-being, proximal stressors mediating their relationship, and resilience factors moderating the effect of distal and proximal stressors on psychological well-being. Grey arrows reflect a negative relationship to the outcome.

The gender minority stress model (GMSM) integrates the findings of the higher prevalence of mental problems with the permanent stigmatization in transpersons [16]. The model is based on the premise that the experiences of stigmatization take the form of a specific, so-called minority stress, which in turn affects the state of health of the transperson concerned (Figure 1). Minority stressors are divided into two different types, distal stressors and proximal stressors. Distal stressors are stressors that are caused by an external source and proximal stressors refer to internal and subjective thoughts and processes within a transperson. However, transpersons are typically confronted with various forms of minority stressors, which can interact with each other. Thus, negative effects caused by minority stress are caused by both individual factors and their interactions. In addition to the description of minority stress effects, two resilience factors are considered within the GMSM. These act on a group-specific (not individual) level and can help to minimize the negative consequences of stigmatization.

In the past, several studies have examined the applicability of GMSM to the development of depressive symptoms in transpersons [5, 7, 8, 18–21]. Depending on the research's focus, different conclusions have been drawn. While studies examining the association between distal minority stressors and depressive symptoms have supported the validity of the GMSM [7, 8, 18–20, 22–26], results of studies focused on the relationship between depressive symptoms and proximal minority stress factors [5, 18, 21] or on the relationship between depressive symptoms and the resilience factors [5, 19–21, 27–29] were less clear. However, it is difficult to compare results across different studies due to different factors. First, previous studies have not consistently standardized minority stressors and resilience factors and have not all used the same instruments, complicating comparisons of collected data between studies. Second, previous studies have neglected to assess if a moderated mediation is taking place by only focusing on partial aspects of GMSM. Moderated mediation is when proximal stressors mediate the relationship between distal stressors and mental health and resilience factors moderate the relationships between distal stressors and mental health and proximal stressors and mental health (see Figure 1). Thus, neglecting to assess all aspects of GMSM inhibits the ability to determine the validity of the overall model.

The aim of this study was to utilize the GMSM to explore the effects of stigmatization on the mental health of transitioned transpersons. In particular, the relevance of each individual factor (i.e., distal minority stress, proximal minority stress, and resilience) and the impact of their interactions were examined using a validated minority stress measurement tool. This holistic and methodologically founded approach overcomes previous research limitations and contributes to a better understanding of stigma-related processes and mental health outcomes. This study focused on transitioned transpersons, because pretransitioned transpersons and transpersons in medical transition are faced with other stressors related to the initiation or completion of the transition (e.g., hormonal side effects and complications with surgery), which do not meet the specific definition of minority stressors [16]. The exclusion of transpersons before and during medical transition in the study helped ensure that there would be no interaction between transition-related stressors and minority stressors. Furthermore, this study concentrated on depressive symptoms for the mental health outcome, as depressive disorders crucially contribute to the global burden of disease of transpersons [30].

In this study, we defined three hypotheses: firstly, there is a positive correlation between distal stressors and depressive symptoms and proximal stressors and depressive symptoms; inversely, there is a negative correlation between resilience factors and depressive symptoms; secondly, all minority stress factors contribute substantially to the explanation of depressive symptoms; thirdly, proximal stressors mediate the relationship between distal stressors and depressive symptoms; resilience factors moderate the relationships between distal and proximal stressors and depressive symptoms.

## 2. Methods

### 2.1. Patients and Procedures

The study was a multicentric collaboration of four Swiss hospitals specialized in treating transpersons: Basel and Zurich (psychiatric, endocrine, and surgical interventions), Bern (endocrine and surgical interventions), and Olten (psychiatric interventions). To maximize the number of potential participants, members of the transcommunity were recruited through Swiss transorganisations.

Transpersons were included in the study if they met the following criteria: German-speaking, at least 18 years of age, and self-identified as transitioned. For this study, patients were characterised as transitioned if they were not currently attending transspecific psychotherapy/counselling, have not taken hormone therapy for at least one year, or did not plan to have any surgical interventions within the next year. Transpersons were excluded if they were in the process of medical transition or transrelated counselling.

Transpersons treated in the four hospitals were recruited via postal notifications and asked to complete either the paper-pencil or online version of the questionnaire battery. Transpersons recruited by the transorganisations could only complete the questionnaire online. Participants gave informed consent before proceeding to fill out the questionnaires and data was anonymized. The Ethics Committee Northwest and Central Switzerland approved this procedure.

### 2.2. Measures

#### 2.2.1. Gender Minority Stress

Gender minority stress was assessed by the validated* Gender Minority Stress and Resilience Measure* (GMSR), which includes four distal minority stress factors, three proximal minority stress factors, and two resilience factors [17]. The measure contains 58 items, with five to nine items per factor and each factor functioning as scale. For the distal factors “gender-related discrimination” (“I have experienced difficulty getting identity documents that match my gender identity”), “gender-related rejection,” and “gender-related victimization,” the categories “never,” “yes, before age 18,” “yes, after age 18,” and “yes, in the past year” items are scored 0 for “never” and 1 for any other category. For the items of the factors “nonaffirmation of gender identity” (“I have to work hard for people to see my gender accurately”), “internalized transphobia” (“Because of my gender identity or expression, I feel like an outcast”), “negative expectations,” “nondisclosure,” “community connectedness” (“I feel connected to other people who share my gender identity”), and “pride” items responses ranged from “strongly disagree” to “strongly agree” with the corresponding score from 0 to 4. For the distal and proximal scales, a higher value signified higher stigmatization, and inversely, a higher value represented higher resilience.

As there was no German version of the questionnaire, the GMSR was translated into German and back to English (with the permission of the developer) to ensure the quality of the translation. The German translation was examined for readability and content. The obtained Cronbach alphas were comparable to the original paper [17] and ranged from acceptable (*α* = .71 for “nondisclosure”) to excellent (*α* = .93 for “negative expectations”). However, this study resulted in an unacceptable *α* = .48 for “gender-related discrimination,” whereas the original paper obtained questionable *α* = .61.

#### 2.2.2. Depressive Symptoms

Depressive symptomatology was assessed by the* Allgemeine Depressionsskala* (ADS-K) [31], which is the German equivalent to the* Center of Epidemiologic Studies Depression Scale* (CES-D) [32]. The ADS-K consists of 15 items, which can be answered on a Likert scale ranging from 0 to 3 with the corresponding answers “rarely or none of the days,” “some or a little of the time,” “occasionally or a moderate amount of time,” or “most or all the time.” The score of every item is summed to obtain one score representing the severity of depressive symptoms. Participants look one week back and self-report if they experienced symptoms associated with depression such as difficulty concentrating, feeling depressed, or having a restless sleep. A higher score indicates more depressive symptomatology. Previous analyses have demonstrated that the ADS-K had good to very good reliability and validity [31]. The Cronbach's alpha for this study was *α* = .94.

#### 2.2.3. Sociodemographics

The extensive sociodemographic survey was thematically divided into three sections: general, transspecific, and transition-specific. In the general section, participants self-reported their age, place of residence, current living arrangement, sexual orientation, relationship status, highest education, and current occupational situation. Experienced gender, gender assigned at birth, and preferred gender label were considered transspecific variables. Gender was added as a new variable and included experienced gender and gender assigned at birth. We defined a transfeminine person as an individual that identified as female but was assigned as a male at birth, a transmasculine person as an individual that identified as male but was assigned as a female at birth, and a gender nonbinary person as an individual that identified as between male and female gender or identified as having no gender, independently of their sex assigned at birth. Transition-specific variables assessed psychological/psychiatric evaluations as well as hormonal and surgical interventions.

### 2.3. Statistical Analyses

For the first hypothesis, bivariate Spearman correlational analyses between the ADS-K score and each gender minority stress factor were conducted [33]. Effect sizes were evaluated following Cohen's guidelines [34].

To test the second hypothesis, a multiple hierarchical regression was performed. Preliminary data analysis revealed a lack of homogeneity of variance and normality of the residues [35]. A Cox-box transformation was conducted in order to counteract the missing prerequisite for multiple linear regression [35]. Control variables for the linear model were selected if they significantly correlated with the ADS-K score and granted sufficient statistical power for the model. Categorical variables were dummy coded: for gender, transfeminine was chosen as the reference category, as the literature reports a higher vulnerability for depressive symptoms in this group [36]. Equivalently, unemployed was chosen as reference category for occupational status [37].

A moderated mediation analysis was conducted to check the last hypothesis [38]. To have sufficient power to conduct the analysis, gender minority stress and resilience factors were summed to obtain the combined variables' distal stress, proximal stress, and resilience. All statistical procedures were conducted using the software IBM SPSS Statistics for Windows [39] and the macro PROCESS [40].

## 3. Results

### 3.1. Participant Characteristics

Data from 143 people were analysed in this study. The age range of the participants was 18–75 years with a mean of 45.2 years (SD = 18.2 y). Most participants were transfeminine (52%) and labelled themselves as “transgender,” “transsexual,” or “transwoman.” At the same time, 30% of the participants defined themselves as transmasculine and 18% as nonbinary. The nonbinary group described themselves as “agender,” “genderfree,” “neutrois,” “genderfluid,” “genderqueer,” or “nonbinary-gender.” When age was split up by gender, a bimodal age distribution became evident, where transfeminine persons (M = 51.5, SD = 17.1) were significantly older in comparison to transmasculine persons (M = 36.0, SD = 12.8; *F* = 10.32, *p* < 0.01). Nonbinary persons had a mean age of 42.2 years (SD = 24.4), which was between the mean ages of the other groups.

The relationship status between groups varied significantly. Participants with binary genders mainly stated that their relationship status was single (transfeminine: 53.9%/transmasculine: 42.5%) or in a relationship (transfeminine: 43.4%/transmasculine: 57.5%), but only 28% of the nonbinary persons were in a relationship and 44.0% stated to be single. In contrast, 28% of this group described themselves as being in an “other” form of relationship (e.g., open or polyamorous network).

In regard to employment status, most transfeminine persons were active on the labour market either employed (35.5%) or self-employed (19.7%). While 19.7% reported to be unemployed, 25.0% found themselves in another situation (e.g., studying, being retired, or getting disability pension). In the transmasculine group, 61.0% reported to be employed and 12.2% to be self-employed; only 7.3% were unemployed and 19.5% were facing another situation. Only 25.0% of nonbinary persons were employed and 16.7% reported to be self-employed. Unemployment in nonbinary persons was 20.8%, while 37.5% found themselves in another situation. More information on the participants' characteristics can be found in Jellestad et al. [15].

Depressive symptomatology varied among the different gender groups (*F* = 5.98, *p* < 0.01). Nonbinary persons exhibited a significantly higher ADS-K score (M = 18.04, SD = 10.17) compared to transfeminine (M = 10.76, SD = 7.80) and transmasculine (M = 12.59, SD = 10.16) persons.

Similarly, there were significant gender specific differences in the scales “gender-related discrimination,” “nonaffirmation of gender identity,” “internalized transphobia,” and “pride” of the GMSR (Table 1).

### 3.2. Correlational Analyses

To check the first hypothesis, correlations between the ADS-K score and each gender minority stress and resilience factor were conducted (Table 2). Accordingly, each distal and proximal factor exhibited significant positive correlations between depressive symptoms and gender minority stress factors with medium to strong effects (ranging from *r* = .30 for gender-related victimization to *r* = .52 for nonaffirmation of gender identity). Resilience factors did not yield clear results. Even though the correlation between community connectedness and depressive symptoms revealed a small significant negative effect, pride and depressive symptoms did not have a significant correlation.

### 3.3. Regression Analyses

For the second hypothesis, a hierarchical multiple regression model was calculated with the normalized ADS-K score as a dependent variable. Since gender and occupational status significantly correlated with the ADS-K score, they were used as control variables. The variable highest education also correlated significantly with the ADS-K score, but issues concerning the power of the model led to rejecting that variable as control variable. In the first step, the control variables age, gender, and occupational status were inserted to the model. Model 1 explained 21% of the variance (*R*^2^ = .21, *F* = 4.62, *p* < 0.001), with the variables nonbinary (*β* = .21, *p* = 0.030), self-employed (*β* = −.33, *p* = 0.003), and employed (*β* = −.40, *p* = 0.002) contributing individually to the explanation of variance. In the second step, distal factors were added to the model. Model 2 explained an additional 23% of the variance (*R*^2^ = .44, *F* = 7.97, *p* < 0.001) with the variables self-employed (*β* = −.23, *p* = 0.018), age (*β* = −.18, *p* = 0.027), gender-related rejection (*β* = .20, *p* = 0.042), and nonaffirmation of gender identity (*β* = .45, *p* < 0.001) contributing individually to the explanation of variance. In the third step, proximal factors were added to the model. Model 3 additionally explained 6% of the variance (*R*^2^ = .50, *F* = 7.51, *p* < 0.001) with the variables self-employed (*β* = −.22, *p* = 0.025), nonaffirmation of gender identity (*β* = .31, *p* = 0.005), and internalized transphobia (*β* = .25, *p* = 0.005) explaining variance individually. For the last step, resilience factors were added to the model. Model 4 could only explain an additional 1% of the variance (*R*^2^ = .51, *F* = 6.63, *p* < 0.001) (Table 3).

### 3.4. Mediation-Moderation Analysis

**Figure 2:**
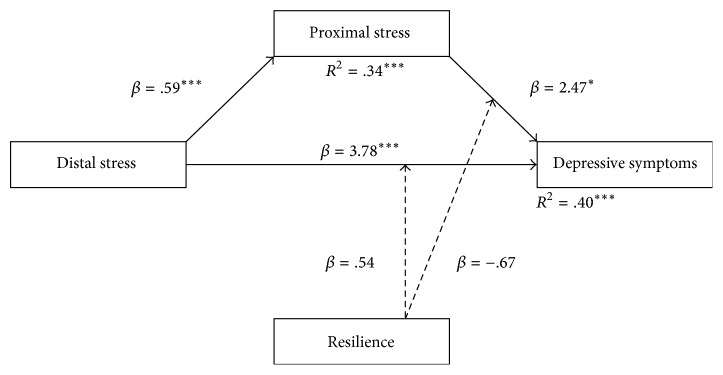
Path model of the direct and indirect effects of proximal and distal stress within the GMSM. Dashed lines represent nonsignificant results. ^*∗∗∗*^*p* < .001; ^*∗*^*p* < .05.

Distal stress had a significant direct effect on depressive symptoms (*β* = 3.78, *p* < 0.001) and proximal stress had a significant indirect effect on depressive symptoms (*β* = 2.47, *p* < 0.05). The moderation of resilience did not yield significance (Figure 2).

## 4. Discussion

This study aimed to expand research on the health consequences of gender minority stigmatization in transpersons. For the first time, the relationships between gender minority stressors, resilience-promoting factors, and depressive symptoms in transpersons were examined simultaneously and in a comprehensive manner using the GMSM [16]. Thus, this research facilitated exploration of the scarcely studied Swiss transpopulation and established an intercultural validity of the proposed biopsychosocial interactions between stigmatization experiences and mental health problems.

### 4.1. Validation of the Gender Minority Stress Model

The overall results indicate the validity of the GMSM. Firstly, distal stressors were highly associated with depressive symptoms. This finding is in line with the large body of previous research about gender-related discrimination, rejection, and victimization [7, 18, 20, 25, 26]. Interestingly, this result confirms the results from the only two studies investigating the effects of nonaffirmation of gender identity [23, 41]. It further indicates that the difficulty for society to recognize the transpersons' gender results in significant negative long-term impact for this group. Perhaps the relationship between society's perception and the transperson's gender is particularly strong in transitioned transpersons. It is possible that the probability of experiencing interpersonal stigma decreases due to the type of GAI undertaken. The problems arising from the nonaffirmation of the own gender (e.g., having to explain one's own gender repeatedly) are difficult to avoid by performing hormonal or surgical measures. Future studies should definitely take the transition time axis more into account and consider the respective minority stressors differently depending on the transition situation. However, comparing results between studies that have investigated distal stressors is problematic because they have used different conceptualizations of the different stressors. To advance understanding of distal factors, future research should also use validated instruments for the assessment of gender minority stressors.

Secondly, proximal stressors also had the expected association with depressive symptoms. Importantly, internalized transphobia as proximal stressor was strongly associated with depressive symptoms. Our results confirm previous research on proximal stressors [5, 21, 41]. Comparisons to other proximal factors cannot be drawn, as there are no existing published studies. At the same time, studies on the lesbian, gay, and bisexual (LGB) population show that constant confrontation with stigmatizing messages leads to internalization, which, in turn, negatively affects the health status of the person concerned [42, 43], thus indicating that gender and sexual minorities seem to be affected by similar stressors of the GMSM.

However, limitations on the validity of the GMSM should be mentioned. First, the resilience factors in general and particularly pride are limitations of the model. Pride did not significantly correlate with depressive symptoms, rejecting the model's assumptions. To our knowledge, only one other study has assessed pride in the context of stigmatization and mental health [5]. That study used the same questionnaire items to assess pride as this study and its findings aligned with the results of this current study. However, pride was handled as subscale of internalized transphobia in the previous study. So, one may ask if pride is a factor separate from internalized transphobia or if pride is the same construct (positive pole) as internalized transphobia (negative pole) and therefore not contributing significantly in explaining variance. There is some evidence in the literature for both the first [18] and second [5] interpretations. In studies on sexual minorities, pride is a well-established resilience factor [44], so it is assumed that similar mechanisms apply to transpersons. Yet, in contrast to transpersons, LGB persons are able to conceal their sexual orientation from public view, so showing pride becomes an active process that can be measured independently of sexual orientation. This situation is different for transpersons in that they do not want to (and cannot) conceal their gender identity from their environment. Therefore, gender identity and pride interact and mix with each other in such a way that objective and individual measurement of each category is more difficult. Future investigations in the GMSM field must take these concerns into account and suggest a more precise definition and measurement of resilience factors with regard to transpersons.

### 4.2. Predictive Factors

In terms of predictability of the GMSM, this study found primarily distal stressors (nonaffirmation of gender identity) and proximal stressors (internalized transphobia) to contribute to depressive symptoms. This is an interesting finding, as both factors are not related to interpersonal stigma level. While the “nonaffirmation of gender identity” items are more related to structural stigma experiences, the “internalized transphobia” questions clearly indicate a self-stigmatization of the transparticipants. This means that although the body of literature focuses on interpersonal stigmas, such as discrimination and victimization [18, 20, 25, 26], it seems that their importance is diminished in this posttransitioned population.

From a clinical perspective, the relationship between distal experiences of nonaffirmation of gender identity and long-term development of depressive symptoms are well understood. Gender dysphoric states arise from both intrinsic differences (between one's own gender identity and the sexually marked body) and extrinsic differences (between mentally experienced and socially committed sex) [45]. By means of medical transition and initiation of the first GAI (e.g., hormonal treatment and gender affirming surgeries), transindividuals initially primarily contribute to the reduction of the intrinsic, and occasionally of the extrinsic, gender dysphoric source. Currently, there has been very little discussion on how these steps contribute to improving mental health and quality of life of transpersons [46].

However, in cases where there is no reduction of extrinsic suffering, gender nonaffirmation of the person concerned acquires a special meaning. It has been argued that for a “successful” affirmation of gender identity, the perception of one-self and of other persons' perception of that person needs to be congruent [47]. If this congruence is undermined, the nonaffirmation of their own gender identity can have long-term detrimental effects on a transperson's well-being. Due to the pressure to conform to a binary gender system, it is not surprising that some posttransitioned transpersons are satisfied after their first medical transition steps and seek further GAI such as facial feminization. According to our data, this dynamic is particularly evident in nonbinary transpersons. The mix of pronounced binary stigma and lack of suitable GAI is strongly related to the high level of depression in this population.

Furthermore, the relationship between internalized transphobia and depressive symptoms found in this study fits well into the existing knowledge body of the GMSM [5, 18, 21, 41]. Once again, it is important to note that the positive effects of GAI do not appear to affect all subsequent problems of stigmatization to the same extent. Therefore, it is important that individuals who exhibit a high degree of self-stigmatization do not exclusively perform somatic treatments to minimize their gender dysphoric symptoms. In order to strengthen their own transidentity, these individuals should be in contact with transcommunity-based care and psychotherapeutic services [48, 49].

In addition to all of the stigma-related factors, the control variable occupational status (with the category “unemployed”) also reached predictive significance. The association between unemployment and depressive symptoms is well established in other populations as well [37]. In this sense, the investigated Swiss transpopulation, which has an unemployment rate four times higher than the general population [50], seems to confirm this negative correlation.

### 4.3. Moderating/Mediating Effects of Stigma

Findings suggesting the occurrence of a moderated mediation for the GMSM are partially supported. For distal and proximal stressors, there was a mediation of proximal stressors between distal stressors and depressive symptoms. This study partly confirms the few previous studies examining the mediating effects of gender minority stressors. In a study by Breslow et al. [21], internalized transphobia did not significantly mediate, but they assessed a construct called stigma awareness (consisting of negative expectations and nondisclosure) that did mediate the relationship between distal stressors and depressive symptoms. Since we did not distinguish between individual proximal stressors to assess the mediation, we cannot say how the different proximal stressors contributed to the mediating effect. Our hierarchical linear regression analysis indicated that internalized transphobia may be the most important proximal stressor.

However, the resilience factors failed to confirm the model's assumed moderation of the relationship between distal and proximal stressors and depressive symptoms (there were no significant interactions). This raised the question of what were the resilience factors influencing, since they explained only 1% of the variance. Based on previous studies that have assessed community connectedness, it is clear that the assumed relevance of resilience factors is usually not confirmed [19, 27] and the validity of using community connectedness as a resilience factor should be further examined. Using community connectedness as a resilience factor can have a protective effect on some transpersons [28, 29], whereas it has a pejorative effect on other groups [21], suggesting that other mechanisms are involved with this resilience factor. Similar to handling issues with assessing the effects of pride as mentioned above, future studies should use a more appropriate operationalization of the “community connectedness” concept.

### 4.4. Strengths and Limitations

A substantial limitation of this study is the cross-sectional design, which only allows us to draw correlational, not causative, conclusions. In light of a better understanding of the GMSM, future studies should address this limitation by considering a longitudinal study design. Another aspect to consider is that study participants were asked to concentrate on a questionnaire for 45 minutes. This aspect could have jeopardized the generalizability of the study, as transpersons with severe mental health problems may not have had the cognitive ability to concentrate for that amount of time and were therefore underrepresented. The last limitation concerns two issues with operationalization. Firstly, for this study, mental health was only assessed through self-assessment of depressive symptoms. Even though we used a validated instrument, it only serves as screening test, so a prevalence estimate of depressive disorders could not be calculated. Secondly, the gender-related discrimination, rejection, and victimization scales were retrospectively answered (before age of 18, after age of 18, and in the last year). Since it has been suggested that recent events of interpersonal stigmatization are more likely to affect a transperson's mental health [25], the operationalization of these scales may not be accurate and should be changed.

Despite the limitations, the major strength of this study was that it approached the GMSM systematically with all proposed stressors and resilience factors. Furthermore, this is the first report to record the long-term stigmatization experiences of a considerable number of nonbinary transpersons in Central Europe.

## 5. Conclusions

In summary, the GMSM provides a heuristic approach when examining the impact of stigmatization on the mental health of transpersons. However, the proposed resilience factors should be revised, as they do not exhibit a consistent moderating effect. Future studies working on GMSM should improve the operationalization and, consequently, the measurement of resilience factors. From a clinical perspective, the history of experiences with stigmatization should be given high priority when in contact with transitioned transpersons. It is also important to note that structural and self-stigmatization episodes frequently occur in this group. In cases where these stigmatization experiences are the basis for the initiation of further GAI measures, especially transpersons exhibiting a high degree of self-stigmatization, transpersons should be supported by a community-based services or psychotherapy.

## Figures and Tables

**Table 1:** Descriptive statistics of the gender minority stress scales divided by gender and ANOVA results.

Scale	Transfeminine M (SD)	Transmasculine M (SD)	Nonbinary M (SD)	*F* (df)	*p*
Gender-related discrimination	2.19 (1.77)	3.14 (2.20)	3.58 (2.55)	5.48 (2,137)	**0.005**
Gender-related rejection	3.19 (2.57)	3.67 (3.71)	4.52 (3.42)	1.69 (2,133)	0.188
Gender-related victimization	1.99 (2.42)	2.69 (2.79)	2.80 (2.93)	1.44 (2,139)	0.241
Nonaffirmation of gender identity	6.69 (5.82)	4.95 (5.97)	14.68 (6.84)	21.95 (2,138)	**0.001**
Internalized transphobia	6.34 (6.78)	10.38 (8.85)	7.88 (5.44)	4.00 (2,134)	**0.020**
Negative expectations	12.75 (9.20)	12.53 (7.84)	16.22 (7.54)	1.65 (2,133)	0.197
Nondisclosure	7.72 (4.61)	8.37 (4.26)	10.32 (5.17)	2.72 (2,136)	0.070
Pride	17.15 (7.95)	12.46 (6.89)	16.44 (7.02)	5.34 (2,138)	**0.006**
Community connectedness	11.50 (4.53)	12.14 (3.15)	11.68 (4.16)	.34 (2,140)	0.718

**Table 2:** Correlational analyses between the ADS-K score and different gender minority stress and resilience factors.

GMSR factor	ADS-K	*p*
Distal stress factors		
Gender-related discrimination	.39	<0.01
Gender-related rejection	.43	<0.01
Gender-related victimization	.30	<0.01
Nonaffirmation of gender identity	.52	<0.01
Proximal stress factors		
Internalized transphobia	.42	<0.01
Negative expectations	.47	<0.01
Nondisclosure	.32	<0.01
Resilience factors		
Pride	−.13	0.14
Community connectedness	−.22	<0.01

**Table 3:** Model 4 of the hierarchical linear regression with the dependent variable ADS-K (normalized).

Scale	Unstandardized coefficient		Standardized coefficient	*F*	*R* ^2^
	*b*	SE	*β*		
Final model				6.63^*∗∗∗*^	.51
Intercept	12.62	3.78			
Age	−.01	.04	−.01		
Transmasculine^a^	−1.25	1.73	−.06		
Nonbinary^a^	.61	2.02	.03		
*Self-employed*^b^	−5.09	2.33	−.21^*∗*^		
Employed^b^	−3.23	2.03	−.18		
Other occupation^b^	−1.33	2.06	−.07		
Gender-related discrimination	.27	.43	.06		
Gender-related rejection	.37	.31	.12		
Gender-related victimization	−.33	.35	−.09		
*Nonaffirmation of gender identity*	.42	.14	.32^*∗∗*^		
*Internalized transphobia*	.28	.11	.23^*∗∗*^		
Negative expectations	.11	.10	.11		
Nondisclosure	.03	.17	.01		
Pride	.02	.11	.01		
Community connectedness	−.24	.18	−.11		

*Notes*. ^*∗∗∗*^*p* < 0.001; ^*∗∗*^*p* < 0.01; ^*∗*^*p* < 0.05; ^a^reference category: transfeminine; ^b^reference category: unemployed.
